# Supraclavicular Irradiation Induces Lymphedema in Breast Cancer Patients Treated with Axillary Lymph Node Dissection and Taxane-Containing Chemotherapy

**DOI:** 10.1155/2024/3250143

**Published:** 2024-07-31

**Authors:** Nanae Horisawa, Akiyo Yoshimura, Isao Oze, Masataka Sawaki, Masaya Hattori, Haruru Kotani, Ayumi Kataoka, Yuri Ozaki, Kazuki Nozawa, Yuka Endo, Daiki Takatsuka, Ayaka Isogai, Hiroji Iwata

**Affiliations:** ^1^ Department of Breast Oncology Aichi Cancer Center, 1-1, Kanokoden, Chikusa-ku, Nagoya 464-8681, Aichi, Japan; ^2^ Department of Breast Surgery Nagoya City University, 1, Kawasumi, Mizuhocho, Mizuho-ku, Nagoya 467-8601, Aichi, Japan; ^3^ Division of Cancer Epidemiology and Prevention Department of Preventive Medicine Aichi Cancer Center Research Institute, 1-1, Kanokoden, Chikusa-ku, Nagoya 464-8681, Aichi, Japan

## Abstract

**Purpose:**

Breast cancer-related lymphedema (LE) significantly impairs the patients' quality of life. Axillary lymph node dissection (ALND) is a strong risk factor for LE in breast cancer surgery. In addition, postoperative administration of docetaxel (DTX) has been reported to be a risk factor for LE in patients who undergo ALND. Herein, we performed the risk of objective LE after ALND.

**Methods:**

Patients who visited the medical follow-up clinic between 12 November 2018 and 11 January 2019 and at least one year postoperatively were eligible for this study. The risk factors for objective LE according to taxane-containing regimen, radiation therapy, and body mass index and the effects of a taxane-containing regimen followed by supraclavicular irradiation on LE were examined.

**Results:**

A total of 214 patients were included in this analysis, and objective LE was observed in 52 patients (24%). Univariate and multivariate analyses showed that only supraclavicular field irradiation was a statistically significant risk factor for objective LE. In addition, the sequential use of taxane-containing regimens and supraclavicular RT was shown to be a more likely risk factor for LE than ALND alone. We also compared each taxane regimen with supraclavicular RT and found that DTX was more likely to be a risk factor for LE in cases of sequential use of supraclavicular RT than with ALND alone. However, when comparing DTX with supraclavicular RT and PTX with supraclavicular RT directly, there was no statistically significant difference in the risk of objective LE between the two groups.

**Conclusion:**

The risk for LE was more likely to be higher with the sequential use of taxane-containing chemotherapy and supraclavicular field irradiation. Therefore, management of LE is important in these cases.

## 1. Introduction

Breast cancer-related lymphedema (LE) causes pain, oedema of the upper limbs, and functional disability and significantly impairs the patients' quality of life [1]. Therefore, it is important to avoid the risk of LE and provide appropriate management for it. Most lymph nodes or lymphatic ducts from the upper limb nodes are located in the axillary lymph node dissection (ALND) field, where surgeons usually remove lymph nodes for dissection [2]. Performing ALND disrupts the lymphatic system of the upper limbs and decreases the patency of the remaining channels due to scarring [3] LE results from functional overload of the lymphatic system, wherein the lymph volume exceeds the transport capabilities of the system [4]. An increase in interstitial fluid above the normal level results in failure of the lymphatic drainage system, which leads to swelling [5]. Some known risk factors for LE are (1) surgery involving the axilla, (2) body mass index (BMI), (3) infection, (4) chemotherapy, and (5) radiation therapy (RT) [6–9]. In particular, ALND in breast cancer surgery is one of the strongest risk factors for LE, and its incidence has been reported to be approximately 6–50% [6, 7, 10–13].

We conducted a previous study of primary breast cancer patients at our institution to identify differences in subjective and objective LE after breast cancer surgery [11]. Our study clearly showed that subjective and objective assessments of LE differed significantly between patients who underwent sentinel lymph node biopsy (SLNB) and those who underwent ALND. In particular, the incidence of LE in the objective assessment was significantly higher in the ALND group, and ALND was identified as a risk factor for LE. Moreover, we examined other risk factors for LE after breast cancer surgery and concluded that ALND and RT were statistically significant risk factors for LE [11].

Taxane-based chemotherapy has also been suggested to be associated with postoperative LE. Especially docetaxel (DTX) has also been suggested to be a risk factor for LE [14–16]. In addition, postoperative administration of DTX has been reported to be a risk factor for LE in patients who have undergone ALND [10]. Although in our previous study, taxane-based regimens were not associated with risk factors for postoperative lymphedema, we did not distinguish between DTX and paclitaxel (PTX) taxane regimens. Therefore, we performed a secondary analysis of the risk of objective LE after ALND using data from our previous study. The purpose of this analysis was to examine the risk factors for objective LE after ALND according to each taxane-containing regimen, RT, and BMI. In addition, we examined the effects of a taxane-containing regimen followed by supraclavicular irradiation on LE [17].

## 2. Patients and Methods

### 2.1. Patients

A total of 216 patients with primary breast cancer who underwent ALND at our hospital, who visited the hospital for regular medical follow-up between 12 November 2018 and 11 January 2019, were recruited. Of the 216 patients enrolled, 2 patients who received both DTX and paclitaxel (PTX) were excluded. To evaluate objective LE, 4-point arm circumference measurements–metacarpophalangeal joint, wrist, 5 cm distal from the elbow, and 10 cm proximal from the elbow–were performed for both arms. Arm circumference measurements were performed by six investigators who were trained before the study. Objective LE was defined as a difference of at least 2 cm between the affected and unaffected arms or when therapeutic intervention had already been performed for LE. We collected data on patient characteristics from the clinical records.

### 2.2. Postoperative Irradiation

Patients who underwent breast-conserving surgery received 50 Gy postoperative irradiation to the residual breast. Patients considered to be at high risk of recurrence received 50 Gy irradiation to the chest wall and supraclavicular region after mastectomy. The dose and field of postoperative irradiation was determined by the radiation oncologist.

### 2.3. Statistical Analysis

Baseline characteristics were evaluated using Pearson's *χ*^2^ test. Univariate and multivariate analyses were also performed using logistic regression. Statistical significance was set at *P* < 0.05. All data analyses were performed using Stata Ver15.

## 3. Results

### 3.1. Characteristics of the Study Population

A total of 214 patients who underwent ALND were included in this analysis. The median time from surgery was 4 years (range, 1–15). Objective LE was observed in 52 patients (24%). The median age was 54.5 years (range, 31–86).

Neoadjuvant or adjuvant chemotherapy was administered to 186 (86.0%) patients. There were 114 who received a DTX-containing regimen and 55 who received a PTX-containing regimen. There were no statistically significant differences in the administration of DTX and PTX between the objective LE group and the no objective LE group (Table 1). Among all the patients, 133 received RT, of which 93 received supraclavicular radiation.

The clinical stage tended to be higher in the objective LE group than in the no objective LE group, and RT tended to be more frequent in the objective LE group than in the no objective group (Table 1).

### 3.2. Risk Factors for Objective LE

Univariate and multivariate analyses showed that supraclavicular field irradiation was a statistically significant risk factor for objective LE. In contrast, DTX and PTX alone were not statistically significant risk factors for objective LE (Table 2).

The sequential use of a taxane-containing regimen and supraclavicular RT was more likely to be a risk factor for LE than ALND alone (no irradiation and no taxane-containing regimen) (Table 3). Therefore, we compared each taxane regimen with supraclavicular RT and found that DTX was more likely to be a risk factor for LE in cases of sequential use of supraclavicular RT than with ALND alone (Table 4). However, when comparing DTX with supraclavicular RT and PTX with supraclavicular RT directly, there was no statistically significant difference in the risk of objective LE between the two groups (Table 5).

## 4. Discussion

Our study revealed that the most significant risk factor for objective LE after ALND was irradiation of the supraclavicular field and not chemotherapy alone, residual breast irradiation alone, or BMI alone. Radiation can lead to increased scarring and LE, and postradiation scarring can be more pronounced in the postoperative axilla. Several studies have reported that irradiation of supraclavicular fields is a risk factor for LE after ALND [13, 18–20]. Hayes et al. reported that regional irradiation after ALND further increased the incidence of LE [19]. Consistent with this finding, in the current study, we also revealed that residual breast irradiation alone was not associated with a higher risk of LE, but supraclavicular irradiation was found to be a risk factor for LE. This suggests that irradiation of the supraclavicular area increases the risk of LE after ALND. Lymphatic flow from the upper extremities goes to the axillary lymph nodes, which in turn drain to the subclavian lymphatic trunk along the subclavian vein and then to the confluence of the subclavian vein and the internal jugular vein (venous angle). Therefore, the supraclavicular area has abundant lymphatic flow in the upper extremities, and this may have been caused by the disturbance of lymphatic flow secondary to RT of the supraclavicular area, thus leading to LE.

DTX has also been suggested to be a risk factor for LE after ALND. The proposed mechanism by which DTX causes LE is the inflammation of vascular endothelial cells, resulting in abnormal capillary permeability [21, 22]. Additionally, repeated administration of DTX is thought to progress interstitial congestion caused by interstitial fluid, potentially exacerbating lymphatic insufficiency. Furthermore, it has been reported that postoperative transient LE is more likely to occur with taxane regimens than with anthracyclines [23]. However, our study showed that DTX alone was not a significant risk factor for LE after ALND. According to Swaroop et al., DTX is a controversial risk factor for LE because it depends on the timing of diagnosis of LE [15]. Thus, one of the reasons why DTX alone was not a risk factor for LE in our study is that the left-right difference between the affected and healthy sides was measured. Moreover, it has been more than one year since the operation, so at least the effect of temporary oedema caused by DTX could be excluded.

There are few reports indicating PTX as a risk factor for lymphedema compared to DTX [24]. However, given the lack of difference between DTX and PTX with respect to the risk of lymphedema in our study, the difference in risk of lymphedema development with different taxane-containing regimens was unclear from the present results.

Although our study showed that DTX or PTX alone was not a risk factor for LE, the sequential use of supraclavicular irradiation and taxane-containing chemotherapy may increase the risk of LE after ALND. This may be because the increased vascular permeability caused by the taxane regimen can trigger oedema at the site of decreased lymphatic transport capacity and scarring caused by ALND and irradiation of the supraclavicular area. The risk factors for LE are complex, suggesting that a combination of various factors may increase the risk of LE. However, in the comparison of taxane-containing regimens, there was no statistically significant difference between the sequential use of supraclavicular field irradiation with DTX and the sequential use of supraclavicular field irradiation with PTX. This was attributed to the small sample size of each group in this study.

LE is a serious complication in patients with breast cancer, and its prevention has been an area of interest. Studies have shown that early prevention is effective [25, 26]. Recently, Paramanandam et al. reported that prophylactic wearing of sleeves reduced the risk of LE after ALND [27]. For patients who require postoperative supraclavicular irradiation after ALND, breast cancer is more advanced, and adequate systemic treatment and local treatment, including RT, are considered necessary. Therefore, it is important to consider preventive measures to avoid postoperative LE when performing supraclavicular field irradiation following ALND, as mentioned above.

Our previous study, subjective LE was defined as a history of perceived swelling of the affected arm, resulting in subjective LE in about half of the patients after axillary dissection [11]. The previous study also examined the relationship between irradiation and history of chemotherapy and subjective LE, but these were not risk factors. In our study, the same results were obtained. Discrepancies between subjective and objective diagnoses of LE have been reported in the past [28–30], suggesting that subjective LE symptoms do not always correspond to an objective diagnosis of LE. One reason for this is that there are no specific symptoms or criteria for LE, but sensory changes such as numbness, pain, and stiffness after axillary surgery may act as confounding factors when patients perceive these as LE.

There are several limitations in our study. First, we have not identified individual data on the irradiated area and the number of lymph node metastases was not included in this analysis. In our institution, patients with four more axillary lymph node metastases were administered supraclavicular field irradiation. Also, we have not evaluated the relationship between dose or completion of cycles or Relative Dose Intensity (RDI) of taxane-containing regimen and LE. Second, it is not known when the onset of LE occurred after surgery. In addition, one of the main limitations of our current study was the selection bias. The choice of postoperative chemotherapy regimen is dependent on each physician, and some physicians may use PTX to avoid DTX for high-risk patients with LE to avoid LE. However, there was no difference in patient background between the PTX and the DTX groups; therefore, it is unlikely to be a major bias in this study. Moreover, the result that the risk of LE after ALND tended to be higher in the cases of sequential use of supraclavicular field irradiation and taxane-containing chemotherapy than in the cases of ALND alone or sequential use of taxane-containing regimen without supraclavicular irradiation still supports the fact that the risk factors for LE are complex and associated with the above mechanisms. In summary, the risk factors for LE are complex, and there are only a few studies on the risk associated with the sequential use of supraclavicular irradiation and taxane-containing chemotherapy. However, as mentioned above, in the comparison of sequential use of supraclavicular field irradiation and each taxane-containing regimen in this study, there were no statistically significant differences due to the small population size of each group. Thus, further studies are needed to determine whether the risk of LE increases with supraclavicular RT versus DTX with supraclavicular RT. In addition, we did not examine the risk of LE based on whether neoadjuvant or adjuvant administration of taxane-containing chemotherapy was used in this analysis, and this point should be examined in the future.

## 5. Conclusion

The most significant risk factor for the development of objective LE after ALND is supraclavicular area irradiation. The risk for LE was more likely to be higher with the sequential use of taxane-containing chemotherapy and supraclavicular field irradiation. Therefore, management of LE is important in cases of sequential supraclavicular irradiation with a taxane-containing regimen.

## Figures and Tables

**Table 1:** Baseline characteristics.

	Objective LE *n* = 52 (%)	No objective LE *n* = 162 (%)	*P* value
Age			0.04
Median (range)	53 (31–86)	61.5 (35–79)	
<40	1 (1.9)	11 (6.8)	
40–49	10 (19.2)	47 (29.0)	
50–59	14 (26.9)	47 (29.0)	
60–69	11 (21.2)	36 (22.2)	
>70	16 (30.8)	21 (13.0)	
Postoperative year			
Median (range)	4.2 (1.0–16.0)	5.1 (1.0–12.5)	
cStage			<0.01
0	3 (5.8)	15 (9.3)	
IA	8 (15.4)	33 (20.4)	
IIA	10 (19.2)	45 (27.8)	
IIB	12 (23.1)	43 (26.5)	
IIIA	3 (5.8)	15 (9.3)	
IIIB	9 (17.3)	7 (4.3)	
IIIC	6 (11.5)	1 (0.6)	
Unknown	1 (1.9)	3 (1.8)	
Axillary surgery			0.01
Level I + II	38 (73.1)	144 (88.9)	
Level I + II + III	14 (26.9)	17 (10.5)	
Unknown	0	1 (0.6)	
Chemotherapy			0.49
None	5 (4.9)	25 (16.2)	
DTX-containing regimen^※1^	32 (61.5)	82 (50.6)	
PTX-containing regimen^※2^	15 (28.8)	41 (25.3)	
Other regimen^※3^	0	15 (9.3)	
Radiation therapy			0.04
None	11 (21.2)	68 (42.0)	
Breast	10 (19.2)	30 (18.5)	
Breast/chest wall + supraclavicular area	30 (57.7)	63 (38.9)	
Unknown	1 (1.9)	1 (0.62)	
BMI			0.03
Low (<18)	3 (5.8)	10 (6.2)	
Intermediate (18-<25)	29 (55.8)	119 (73.4)	
High (≥25)	20 (38.4)	33 (20.4)	

LE, lymphedema; BMI, body mass index. ^※1^DTX-containing regimen: doxorubicin and cyclophosphamide followed by docetaxel or docetaxel and cyclophosphamide. ^※2^PTX-containing regimen: doxorubicin and cyclophosphamide followed by paclitaxel. ^※3^Other regimens: patients received only doxorubicin and cyclophosphamide or combination chemotherapy with cyclophosphamide, methotrexate, and fluorouracil (CMF) or oral fluoropyrimidines (S-1).

**Table 2:** Univariate analysis and multivariate analysis.

	Univariate			Multivariate		
	OR	95% CI	*P* value	OR	95% CI	*P* value
Chemotherapy						
None	1			1		
DTX-containing regimen^※1^	1.87	0.65–5.33	0.24	1.45	0.58–13.27	0.50
PTX-containing regimen^※2^	1.75	0.56–5.43	0.33	1.54	0.51–13.44	0.50
Axillary surgery						
Level I + II	1					
Level I + II + III	3.12	1.41–6.89	<0.01	2.34	0.92–5.91	0.07
Radiation therapy						
None	1			1		
Breast	2.06	0.79–5.37	0.14	1.60	0.63–4.58	0.30
Breast/chest wall + supraclavicular area	2.94	1.36–6.36	<0.01	2.60	1.15–5.90	0.02
BMI						
Low (<18)	1			1		
Intermediate (18-<25)	0.81	0.21–3.14	0.76	1.60	1.15–5.90	0.60
High (≥25)	2.02	0.49–8.23	0.32	3.90	0.73–20.86	0.10

OR, odds ratio; CI, confidence interval; BMI, body mass index. ^※1^DTX-containing regimen: doxorubicin and cyclophosphamide followed by docetaxel or docetaxel and cyclophosphamide. ^※2^PTX-containing regimen: doxorubicin and cyclophosphamide followed by paclitaxel.

**Table 3:** The risk for LE of sequential use of taxane-containing regimen and supraclavicular irradiation.

Treatment		Objective LE		
Chemotherapy	RT	OR	95% CI	*P* value
None	None	1		
Taxane-containing regimen^※1^	None	1.27	0.41–3.89	0.67
Taxane-containing regimen	Breast/chest wall + supraclavicular RT	3.12	1.21–8.03	0.02

^※1^Taxane-containing regimen: doxorubicin and cyclophosphamide followed by docetaxel or docetaxel and cyclophosphamide or doxorubicin and cyclophosphamide followed by paclitaxel.

**Table 4:** The risk for LE of sequential use of each taxane-containing regimen and supraclavicular irradiation.

Treatment		Objective LE		
Chemotherapy	RT	OR	95% CI	*P* value
None	None	1		
PTX-containing regimen^※1^	None	1.97	0.59–6.60	0.27
PTX-containing regimen	Breast/chest wall + supraclavicular RT	2.88	0.87–9.56	0.08
DTX-containing regimen^※2^	None	1.27	0.41–3.88	0.67
DTX-containing regimen	Breast/chest wall + supraclavicular RT	3.97	1.44–10.9	<0.01

RT, radiation therapy; LE, lymphedema; OR, odds ratio; CI, confidence interval. ^※1^PTX-containing regimen: doxorubicin and cyclophosphamide followed by paclitaxel. ^※2^DTX-containing regimen: doxorubicin and cyclophosphamide followed by docetaxel or docetaxel and cyclophosphamide.

**Table 5:** Risk of LE comparing sequential use of supraclavicular RT and PTX and sequential use of supraclavicular RT and DTX.

Chemotherapy	RT	Objective LE		
		OR	95% CI	*P* value
PTX-containing regimen^※1^	Breast/chest wall + supraclavicular RT	1		
DTX-containing regimen^※2^	Breast/chest wall + supraclavicular RT	1.38	0.51–3.69	0.53

RT, radiation therapy; LE, lymphedema; OR, odds ratio; CI, confidence interval. ^※1^PTX-containing regimen: doxorubicin and cyclophosphamide followed by paclitaxel. ^※2^DTX-containing regimen: doxorubicin and cyclophosphamide, followed by docetaxel, docetaxel, and cyclophosphamide.

## Data Availability

The data that support the findings of this study are not publicly available due to privacy or ethical restrictions but are available from the corresponding author upon reasonable request.

## References

[1] Ridner S. H. (2005). Quality of life and a symptom cluster associated with breast cancer treatment-related lymphedema. *Support Care Cancer*.

[2] Ikeda K., Ogawa Y., Kajino C. (2014). The influence of axillary reverse mapping related factors on lymphedema in breast cancer patients. *European Journal of Surgical Oncology (EJSO)*.

[3] Boneti C., Korourian S., Bland K. (2008). Axillary reverse mapping: mapping and preserving arm lymphatics may be important in preventing lymphedema during sentinel lymph node biopsy. *Journal of the American College of Surgeons*.

[4] Brennan M. J., DePompolo R. W., Garden F. H. (1996). Focused review: postmastectomy lymphedema. *Archives of Physical Medicine and Rehabilitation*.

[5] Smith J. (1998). The practice of venepuncture in lymphoedema. *European Journal of Cancer Care*.

[6] Helyer L. K., Varnic M., Le L. W., Leong W., McCready D. (2010). Obesity is a risk factor for developing postoperative lymphedema in breast cancer patients. *The Breast Journal*.

[7] Zou L., Liu F. H., Shen P. P. (2018). The incidence and risk factors of related lymphedema for breast cancer survivors post-operation: a 2-year follow-up prospective cohort study. *Breast Cancer*.

[8] Ferguson C. M., Swaroop M. N., Horick N. (2016). Impact of ipsilateral blood draws, injections, blood pressure measurements, and air travel on the risk of lymphedema for patients treated for breast cancer. *Journal of Clinical Oncology*.

[9] Coriddi M., Khansa I., Stephens J., Miller M., Boehmler J., Tiwari P. (2015). Analysis of factors contributing to severity of breast cancer-related lymphedema. *Annals of Plastic Surgery*.

[10] Akezaki Y., Tominaga R., Kikuuchi M. (2019). Risk Factors for Lymphedema in Breast Cancer Survivors Following Axillary Lymph Node Dissection. *Progress in Rehabilitation Medicine*.

[11] Terada M., Yoshimura A., Sawaki M. (2020). Patient-reported outcomes and objective assessments with arm measurement and bioimpedance analysis for lymphedema among breast cancer survivors. *Breast Cancer Research and Treatment*.

[12] Fu M. R. (2014). Breast cancer-related lymphedema: Symptoms, diagnosis, risk reduction, and management. *World Journal of Clinical Oncology*.

[13] Rebegea L., Firescu D., Dumitru M., Anghel R. (2015). The incidence and risk factors for occurrence of arm lymphedema after treatment of breast cancer. *Chirurgia (Bucur)*.

[14] Hugenholtz-Wamsteker W., Robbeson C., Nijs J., Hoelen W., Meeus M. (2016). The effect of docetaxel on developing oedema in patients with breast cancer: a systematic review. *European Journal of Cancer Care*.

[15] Swaroop M. N., Ferguson C. M., Horick N. K. (2015). Impact of adjuvant taxane-based chemotherapy on development of breast cancer-related lymphedema: results from a large prospective cohort. *Breast Cancer Research Treat*.

[16] Cariati M., Bains S. K., Grootendorst M. R. (2015). Adjuvant taxanes and the development of breast cancer-related arm lymphoedema. *British Journal of Surgery*.

[17] Nozawa K., Takatsuka D., Endo Y. (2022). Impact of tumor progression on survival during neoadjuvant chemotherapy in breast cancer: a cohort study. *Anticancer Research*.

[18] Shaitelman S. F., Chiang Y. J., Griffin K. D. (2017). Radiation therapy targets and the risk of breast cancer-related lymphedema: a systematic review and network meta-analysis. *Breast Cancer Research Treat*.

[19] Hayes S. B., Freedman G. M., Li T., Anderson P. R., Ross E. (2008). Does axillary boost increase lymphedema compared with supraclavicular radiation alone after breast conservation?. *Physics*.

[20] Powell S. N., Taghian A. G., Kachnic L. A., Coen J. J., Assaad S. I. (2003). Risk of lymphedema after regional nodal irradiation with breast conservation therapy. *Physics*.

[21] Semb K. A., Aamdal S., Oian P. (1998). Capillary protein leak syndrome appears to explain fluid retention in cancer patients who receive docetaxel treatment. *Journal of Clinical Oncology*.

[22] Béhar A., Pujade-Lauraine E., Maurel A. (1997). The pathophysiological mechanism of fluid retention in advanced cancer patients treated with docetaxel, but not receiving corticosteroid comedication. *British Journal of Clinical Pharmacology*.

[23] Lee M. J., Beith J., Ward L., Kilbreath S. (2014). Lymphedema following taxane-based chemotherapy in women with early breast cancer. *Lymphatic Research and Biology*.

[24] Aoishi Y., Oura S., Nishiguchi H. (2020). Risk factors for breast cancer-related lymphedema: correlation with docetaxel administration. *Breast Cancer*.

[25] Carretti G., Mirandola D., Maestrini F. (2022). Quality of life improvement in breast cancer survivors affected by upper limb lymphedema through a novel multiperspective physical activity methodology: a monocentric pilot study. *Breast Cancer*.

[26] Hayes S. C., Johansson K., Stout N. L. (2012). Upper-body morbidity after breast cancer: incidence and evidence for evaluation, prevention, and management within a prospective surveillance model of care. *Cancer*.

[27] Paramanandam V. S., Dylke E., Clark G. M. (2022). Prophylactic use of compression sleeves reduces the incidence of arm swelling in women at high risk of breast cancer-related lymphedema: a randomized controlled trial. *Journal of Clinical Oncology*.

[28] Sackey H., Johansson H., Sandelin K. (2015). Self-perceived, but not objective lymphoedema is associated with decreased long-term health-related quality of life after breast cancer surgery. *European Journal of Surgical Oncology (EJSO)*.

[29] Hayes S., Cornish B., Newman B. (2005). Comparison of methods to diagnose lymphoedema among breast cancer survivors: 6-month follow-up. *Breast Cancer Resarch Treat*.

[30] Lopez Penha T. R/, Slangen J. J., Heuts E. M., Voogd A. C., Von Meyenfeldt M. F. (2011). Prevalence of lymphoedema more than five years after breast cancer treatment. *European Journal of Surgical Oncology (EJSO)*.

